# Supplementary material for: Increased Fat Taste Preference in Progranulin-Deficient Mice
Source: Nutrients. 2021 Nov 17;13(11):4125. doi: 10.3390/nu13114125 (PMC8623710; doi:10.3390/nu13114125)
Supplement: Supplementary file 1 [file nutrients-13-04125-s001.zip › nutrients-1406893-supplementary.pdf]

Supplementary table and supplementary figures

To the manuscript:

Increased fat taste preference in progranulin deficient mice

Lana Schumann [1], Annett Wilken-Schmitz [1], Sandra Trautmann [1], Alexandra Vogel [1], Yannick Schreiber [1,2] , Lisa Hahnefeld [1,2], Robert Gurke [1,2], Gerd Geisslinger [1,2,3], and Irmgard Tegeder [1,*]

Supplementary Table S1: Antibodies

Antibody	Host	Dilution	Source	Product #	Type	Target
**CD36/SCARB3**	rabbit	1:200	NovusBio	nb400-144	pab	Human, Mouse
**Cytokeratin 14 (CK14)**	guinea pig	1:200	Antibodies-online	ABIN113455	pab	Human, Mouse
**α-Gustducin (GNAT3)**	goat	1:200	myBioSource	MBS6005782	pab	Mouse, Rat

Supplementary Figures

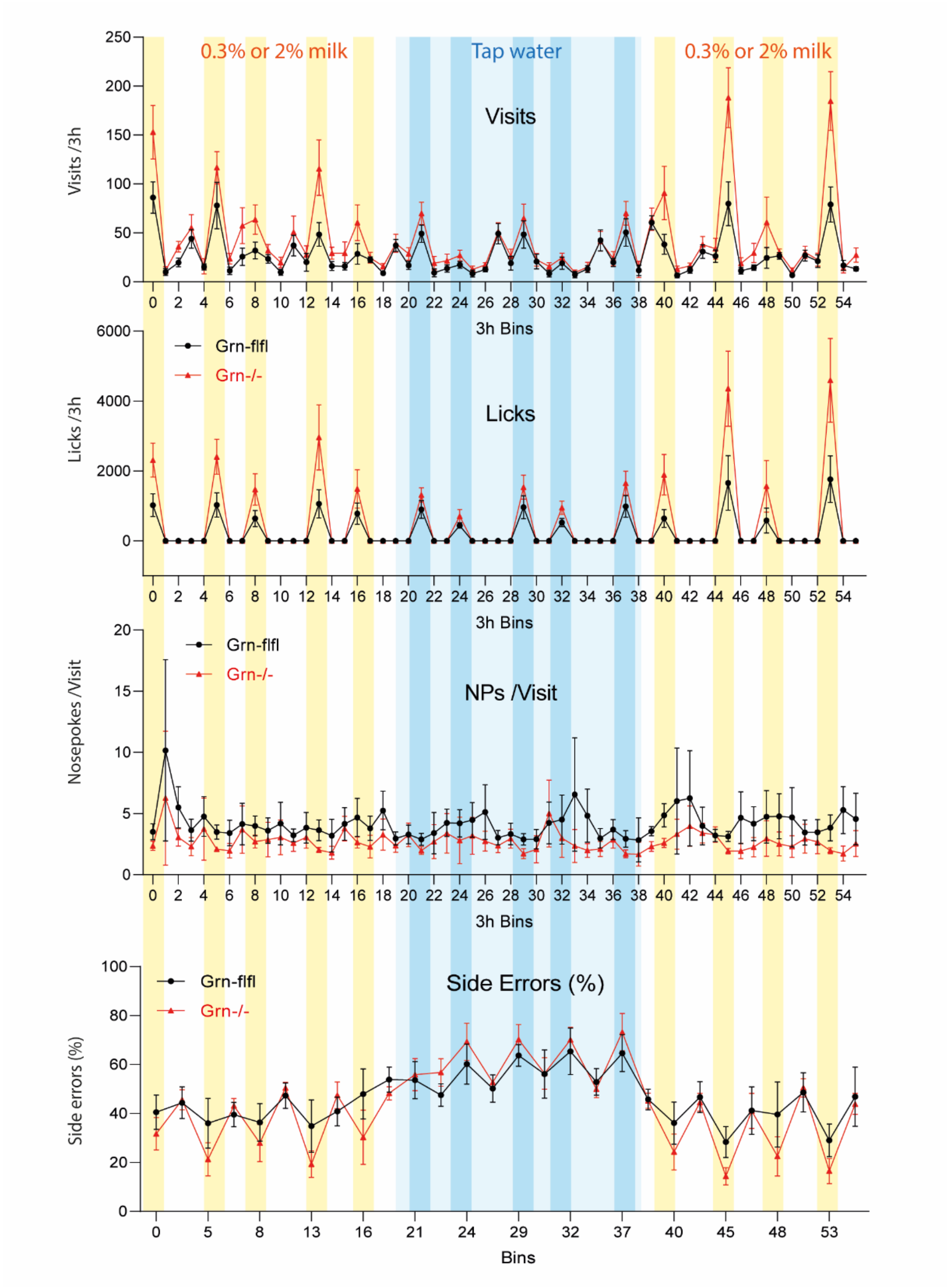

Suppl. Figure S1

Time course of IntelliCage behavior plotted continuously in 3h intervals

Female progranulin knockout (Grn$^{-/-}$) and control mice (Grn-flfl) were housed in two IntelliCages, in each cage n=7-8 per genotype, age 28-37 at the start of the experiment. In the fat taste preference module, mice could freely choose between 0.3% skim milk on one side and 2% milk on the other in each corner. This module was active 2x3 h each day (11-14:00 and 02-0.5:00). In between, doors remained closed (default module), so that licks are zero. After 3 days, the bottles were switched to tap water for 2 days, and back to milk for another two days. Side errors are defined as nosepoke on the skim milk side. The

side definition was maintained in the tap water period, but both bottles contained tap water. The yellow and blue stripes highlight nighttime and daytime behavioral fluctuations.

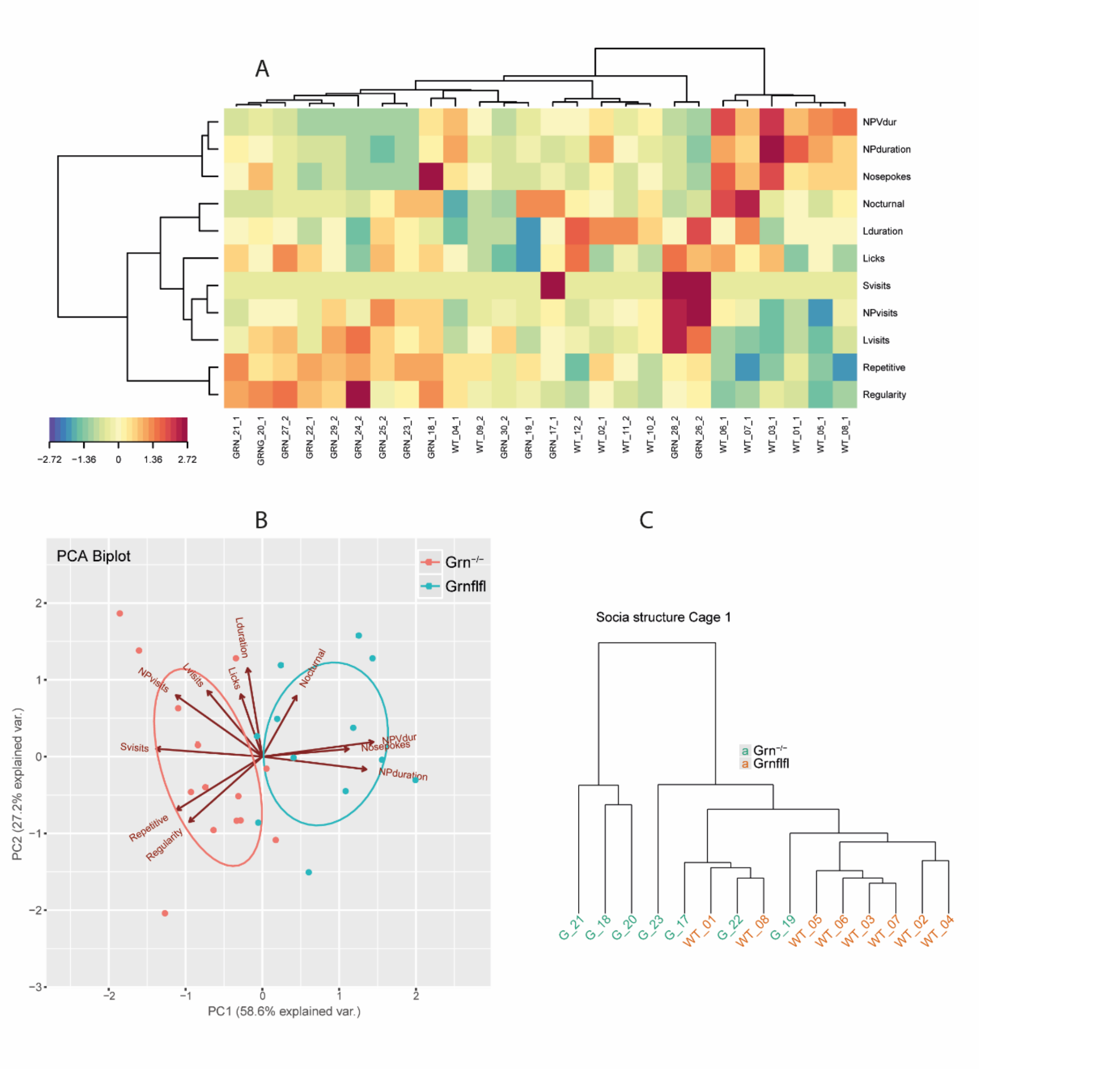

Suppl. Figure S2

Clustering of mice based on IntelliCage behavior, principal component analysis and social structure

A: Heatmap with dendrograms showing the clustering of mice (columns) and behavioral parameters (rows) of IntelliCage behavior. The abbreviations of mice are Grn for $Grn^{-/-}$ and WT for Grn-flfl controls. The mouse number is followed by the cage number. Behavioral abbreviations:

**Visits**	**Visits / h**
**NPvisits**	Visits with Nosepoke without Licks / h
**Lvisits**	Visits with Licks / h
**SVisits**	Visits without Licks and without Nosepokes / h
**NPVdur**	Median duration of Visits with NP w/out Lick (s)
**Nosepokes (NP)**	Mean number of Nosepokes during Visits with NP w/out Licks
**NPduration**	Median duration of such Nosepokes during a Visit (s)
**Licks**	Median number of Licks per Visit
**Lduration**	Median duration of Licking during a Visit (s)
**Nocturnal**	Log(Visit frequency during dark phase / Visit frequency during light phase)
**Repetitive**	Log(sum of observed returns to same corner / sum of expected such switches)
**Regularity**	Sqrt (sum of sq non-diag. transition matrix residuals / sum of non-diag. transition matrix observed values)

B: Principal component analysis (PCA) biplot showing the XY-scatter of mice according to the first two principal components PC1 and PC2. The arrows show the loading. The spheroids show the 95% confidence intervals.

C: Social structure of IntelliCage-1 based on the analysis of the sequence of corner visits, i.e. who followed whom.

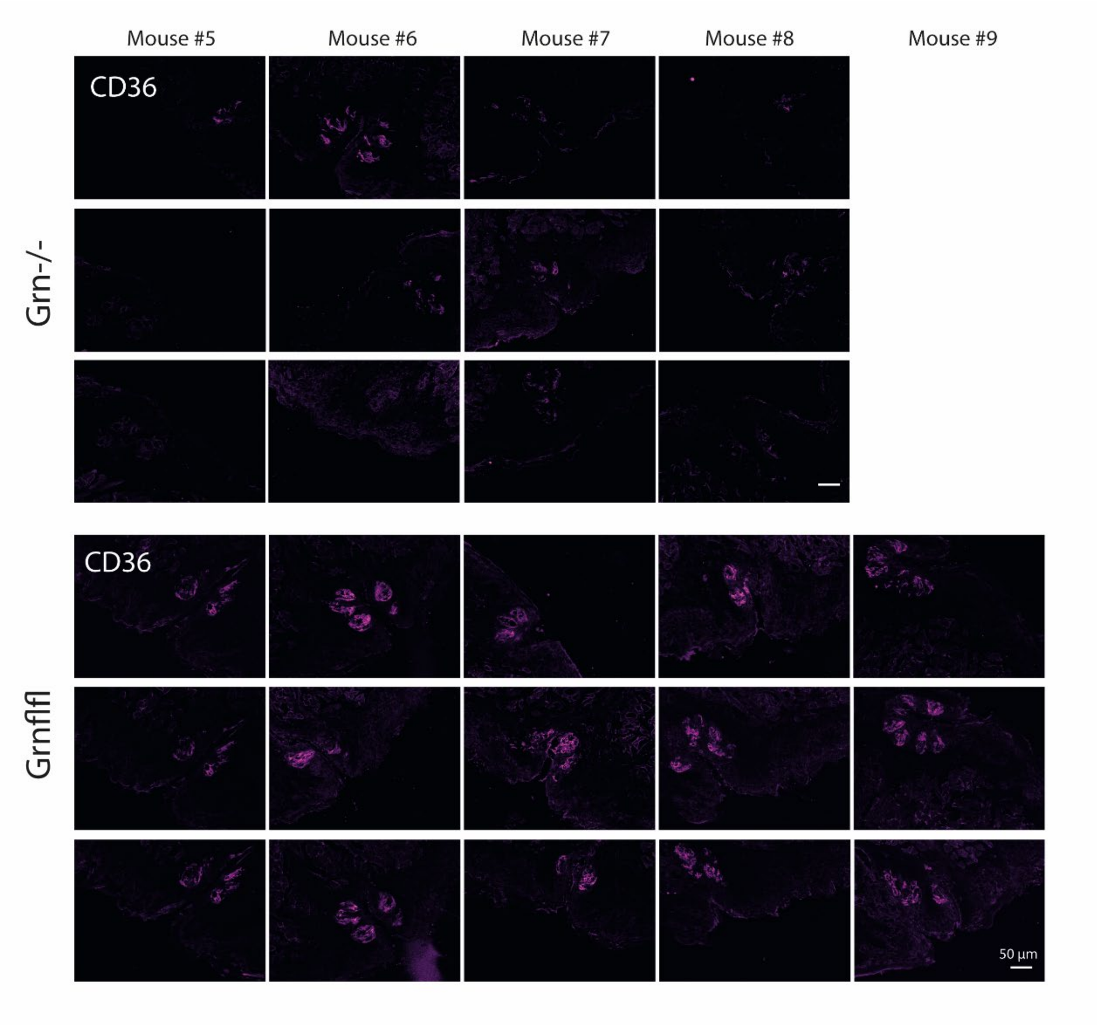

Suppl. Figure S3

Immunofluorescence analysis of CD36 in taste buds

The images show CD36 immunofluorescence in circumvallate papillae of $Grn^{-/-}$ and Grn-flfl mice. Each three sections / views are shown in columns. Mice 1-4 are shown in the main manuscript. Scale bar 50 µm.

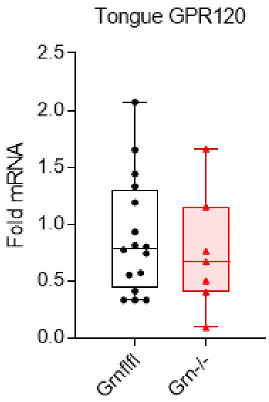

Suppl. Figure S4

Quantitative RT-PCR analysis of GPR120 in the tongue

QRT-PCR analysis of GPR120 in the tongue of $Grn^{-/-}$ and Grn-flfl of the CVP region and the tongue tip Both sites were summarized because the expression of GPR120 was low and some samples had cycle numbers above the upper threshold of 38 cycles. The presented data are of n = 7-8 mice per genotype and triplicate analyses. Data show the fold difference versus the mean of Grn-flfl control mice. EEF was used as housekeeping gene for normalization of the cycle numbers. Data were analyzed according to the ΔΔCt method. Data were compared with unpaired, 2-tailed Student's t-test. There was no difference between genotypes.
